# Fabrication, Microstructure and Plasma Resistance Behavior of Y–Al–Si–O (YAS) Glass-Ceramics Coated on Alumina Ceramics

**DOI:** 10.3390/ma17184585

**Published:** 2024-09-18

**Authors:** Eui Keun Park, Hwan-Yoon Jang, Seo-Yeon Jeon, Kati Raju, Hyun-Kwuon Lee

**Affiliations:** School of Advanced Materials Science and Engineering, Kumoh National Institute of Technology, Gumi 39177, Republic of Korea; czarkhan86@naver.com (E.K.P.); pray330@naver.com (H.-Y.J.); cilcis@naver.com (S.-Y.J.)

**Keywords:** alumina, microstructure, Y–Al–Si–O layer, melt coating, plasma resistance, semiconductor equipment

## Abstract

This study investigates the fabrication, microstructural characteristics and plasma resistance of Y–Al–Si–O (YAS) glass-ceramics coated on alumina ceramics. YAS frits were initially prepared using a melt-quenching method, then homogenously milled and coated onto alumina ceramics. The melt-coating process was conducted at 1650 °C for 1 h. The composition and microstructure of the glass frits and coatings were thoroughly characterized using X-ray diffraction, scanning electron microscopy, and energy-dispersive X-ray spectroscopy. These analyses revealed a dense microstructure with a polycrystalline structure predominantly composed of Y_3_Al_5_O_12_ (YAG) phase and a minor phase of Y_2_Si_2_O_7_. The YAS coatings on alumina revealed a dense layer with strong adhesion to the substrate. Subsequently, the coatings underwent C_4_F_6_/Ar/O_2_ plasma treatment for 1 h. Plasma exposure tests demonstrated that the YAS-coated alumina exhibited significantly better etching resistance compared to uncoated alumina, with minimal surface damage observed on the YAS coating, confirming its protective properties against plasma. The superior plasma resistance of YAS coatings is attributed to the predominance of its YAG phase. This research offers a more stable and cost-efficient solution for protecting ceramics in demanding plasma environments.

## 1. Introduction

Plasma etching is a vital technique in semiconductor manufacturing, crucial for the precise patterning of integrated circuits and electronic devices. This process enables the controlled removal of material from the surface of a semiconductor wafer, achieving high-resolution features and excellent material selectivity [1,2,3,4,5]. These capabilities make plasma etching essential for advancing the miniaturization and performance of semiconductor devices. However, the trend towards miniaturizing electronic integrated circuits, reducing line widths and processing of high-aspect-ratio structures necessitates the use of high-power plasma. Consequently, process equipment parts must possess greater plasma resistance and high-temperature stability to withstand these demanding conditions.

Ceramics play a crucial role in plasma etching chambers, which are integral to the semiconductor manufacturing process. Various ceramic materials, such as alumina, quartz, silicon carbide and aluminum nitride, are commonly used due to their excellent thermal stability, chemical resistance and mechanical strength [6,7,8,9,10,11]. These materials are commonly employed in the form of showerheads, lift pins, insulator pipes, focus rings and confinement rings for different modules of the plasma chamber. The superior properties of ceramics ensure the durability and longevity of these etching chamber components, which are essential for maintaining the precision and efficiency of the plasma etching process. However, contamination from ceramics in plasma etching chambers presents a significant challenge [12,13,14]. During the etching process, ceramic materials can interact with the reactive plasma environment, leading to the release of particulate contaminants. These contaminants can then be deposited on the semiconductor wafers, potentially causing defects and compromising the performance of the final devices.

Mitigating ceramic particle contamination in plasma etching chambers is crucial for maintaining the integrity and performance of semiconductor devices. One effective strategy is the selection and use of high-purity plasma-resistant ceramic materials for chamber components. High-purity ceramics, such as yttria, yttrium fluoride, yttrium oxyfluoride and yttrium aluminum garnet, are less prone to shedding particles under plasma exposure [15,16,17,18,19,20,21]. Different fluorine-based plasma gas compositions including NF_3_, CF_4_ and CHF_3_ are investigated [12,14,17,22,23,24,25] and the results revealed that these yttrium-based ceramics exhibit improved plasma-resistance behavior when compared with alumina and quartz materials. However, high-density yttrium-based materials are cost-ineffective from a commercial standpoint.

Another vital approach involves employing protective coatings on ceramic components to create a barrier that reduces particle generation. For example, Kim et al. deposited Y_2_O_3_ and YF_3_ coatings [17], whereas Jung et al. coated CaO-based silicate glass layers [26] on alumina ceramics and evaluated their plasma resistance behaviors. In another work, Choi et al. reported on the enhanced plasma resistance of quartz after coating with Y_2_O_3_-based silicate glass-ceramic through an aerosol deposition method [27]. Common methods of ceramic coating include physical vapor deposition [17], atmospheric plasma spraying [28], aerosol deposition [27,29], atomic layer deposition [30], chemical vapor deposition [31] and melt-coating [18,32]. Each method offers unique advantages, making ceramic coatings versatile solutions for enhancing durability, corrosion resistance and thermal stability. Among these, melt-coating is a versatile, cost-effective and promising technique for coating different materials. Its advantages include inexpensive equipment, rapid deposition, dense coating layer, thickness and composition control, strong adhesion to ceramics and a desirably smooth surface.

On the other hand, aluminosilicate-based glass-ceramics, including Ca–Al–Si–O, Mg–Al–Si–O, Y–Al–Si–O and RE–Al–Si–O (RE: Rare earth elements) have emerged as promising candidate materials for thermal barrier coatings, plasma etching chambers and high-temperature applications due to their exceptional properties [6,26,27,33,34,35,36,37,38]. Due to their excellent thermal stability, chemical and plasma resistance and adherence properties, Y–Al–Si–O or Y_2_O_3_–Al_2_O_3_–SiO_2_ (YAS)-based glass-ceramics are particularly suited for the harsh conditions of plasma etching environments. These YAS-based coatings can effectively shield the underlying ceramic surfaces from direct exposure to reactive plasma, thereby preventing erosion and the subsequent release of contaminating particles [6,27,38]. Hence, implementing YAS-based coatings offers numerous benefits that enhance the operational efficiency and reliability of plasma etching chambers. Thus, the integration of YAS-based glass-ceramics with melt-coating technology may be considered a feasible and cost-effective approach for contamination control, supporting the stringent quality and performance standards required in modern semiconductor fabrication.

Therefore, in this study, the potential application of a typical YAS-based dense glass-ceramic coating layer on top of alumina ceramics was investigated, expecting a reduction in plasma etching behavior. Initially, YAS frits were prepared via a melt-quenching method at 1650 °C, milled homogenously and coated onto the alumina ceramics. Later, these coatings were subjected to C_4_F_6_/Ar/O_2_ plasma treatment for 1 h. The objective was to evaluate the plasma resistance of alumina with YAS coatings compared to the uncoated alumina substrates, thereby demonstrating their superior plasma resistance behavior. This research provides a more stable and cost-efficient solution to protect ceramics in demanding harsh plasma environments.

## 2. Materials and Methods

To prepare YAS-based frits, high-purity (>99.9%) Y_2_O_3_, Al_2_O_3_ and SiO_2_ powders (Kojundo, Sakado, Japan) are used in the present work. After weighing the raw material powders according to a typical composition of 29 Y_2_O_3_, 33 Al_2_O_3_ and 38 SiO_2_ (mol%), the as-received powders were thoroughly mixed. This composition was chosen in view of its excellent Y_3_Al_5_O_12_ (YAG) and Y_2_Si_2_O_7_ phase-forming ability, which is located at the eutectic point of the Y_2_O_3_–Al_2_O_3_–SiO_2_ phase diagram [39,40]. To ensure a homogeneous distribution of phases in the mixture, ball milling was performed with alumina balls for 4 h using ethanol as the dispersion medium. The mixed slurry was vacuum-dried at 110 °C for 24 h. Later, the obtained powder mixture was placed in a platinum crucible, heat treated at 1650 °C for 2 h in a furnace, and then cooled to obtain YAS-based frits. As-prepared YAS frits were ground thoroughly using planetary ball milling (Pulverisette 6, Fritsch, Idar-Oberstein, Germany), applied in the form of a slurry to the surface of the Al_2_O_3_ base materials and then melt-coated at 1650 ℃ for 1 h. More details about the processing can be found in the literature [26]. Disc-shaped (12 mm diameter and 4 mm thickness) alumina ceramics with a relative density of 99% or higher manufactured using commercial Al_2_O_3_ powder (AL-160SG, Showa Denko, Oyama City, Japan) were used as the base materials in this study. Sintering of alumina ceramics was performed in air using a furnace at 1650 °C for 2 h. All the specimens were polished thoroughly before coating in order to obtain a smooth and homogenous deposition.

The particle sizes of raw powders were investigated using a laser-diffraction particle size analyzer (PSA, Mastersizer 2000, Malvern, UK). The microstructures of the prepared frit and coating layer specimens were observed using a field emission scanning electron microscope (FE-SEM, JEOL, Tokyo, Japan) and the composition analysis of each crystal phase was performed using an Energy Dispersive X-ray spectrometer (EDS) attached to the FE-SEM. An X-ray diffractometer (XRD, Rigaku, Tokyo, Japan) with Cu Kα radiation (λ = 0.15406 nm) was used to analyze the phase structures of the frit and coating layer at room temperature. The frit was powdered and the coating layer was polished smoothly before measurement. Phase fractions were calculated using the integrated intensities of all crystalline and amorphous phases of the measured XRD patterns. Plasma etching tests were performed on specimens with and without YAS coating for 1 h with C_4_F_6_, Ar and O_2_ gasses using TCP-9600PTX (LAM Research Co., Fremont, CA, USA) equipment. The detailed experimental conditions are presented in Table 1. For this purpose, half of the specimens’ surfaces were masked with polyimide tape (Figure S1). The etching rate was evaluated through the weight reduction rate per unit area of the specimens before and after plasma etching. An electronic micro balance with a readability of 10^−4^ g was used to measure the weight of the specimens. The surfaces of all the specimens were polished using diamond paste before plasma treatment.

## 3. Results and Discussion

**Figure 1:**
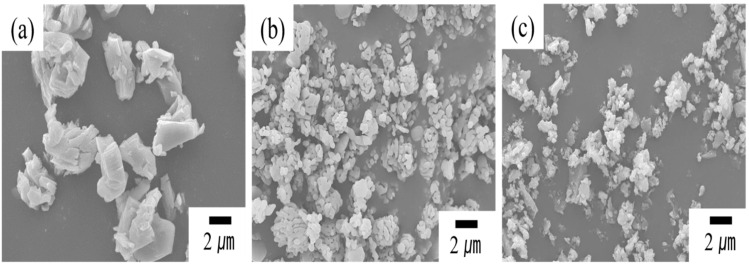
SEM micrographs of the as-received raw powders (**a**) Y_2_O_3_, (**b**) Al_2_O_3_ and (**c**) SiO_2_. These images demonstrate that all the raw powders contain fine and micron-sized particles with irregular morphologies.

Figure 1 presents SEM micrographs of the as-received raw powders Y_2_O_3_, Al_2_O_3_ and SiO_2_. These images demonstrate that all the raw powders contain fine and micron-sized particles with irregular morphologies. PSA analysis (Figure S2) revealed average particle sizes of about 4.4, 2.7 and 0.7 μm, respectively, for Y_2_O_3_, Al_2_O_3_ and SiO_2_ raw powders. These findings are consistent with the observations from the SEM images.

**Figure 2:**
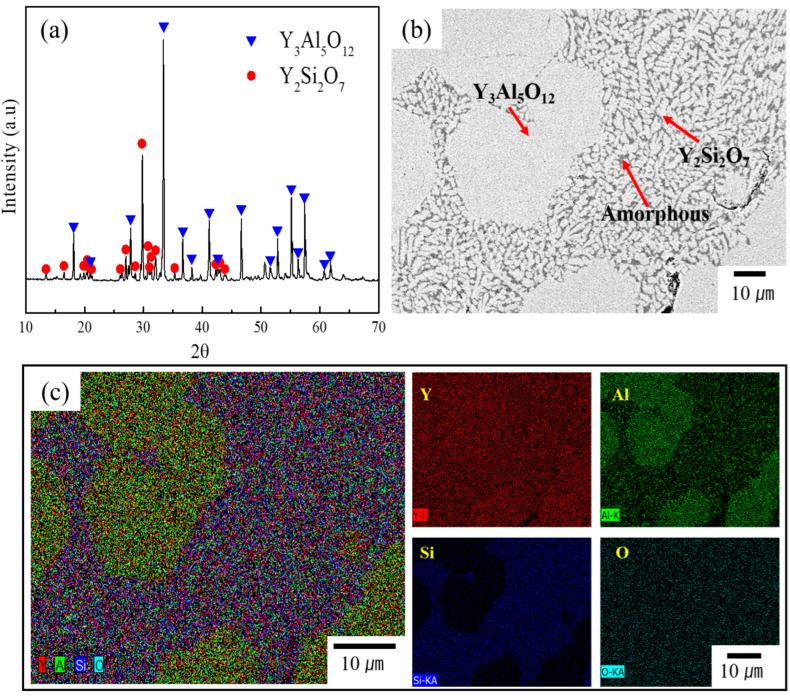
(**a**) XRD patterns of YAS frit after heat treatment at 1650 °C. The controlled heat treatment resulted in the formation of glass-ceramics, which revealed a polycrystalline structure predominantly composed of a major phase of YAG (JCPDS card no:79–1891) [41] and minor phase of Y_2_Si_2_O_7_ (JCPDS card no:42–0168) [41]. (**b**) SEM (BSE mode) image of typical YAS frit, revealing a dense microstructure. (**c**) EDS elemental mapping and elemental distribution of Y, Al, Si and O elements. This figure shows mainly light gray grains with two different morphologies, both large and small. Based on the EDS point analysis, the large grains were identified as the YAG phase and small grains as the Y_2_Si_2_O_7_ phase. The dark gray phase was identified as the YAS amorphous (glass) phase.

Figure 2a shows the XRD patterns of YAS frit after heat treatment at 1650 ℃. The controlled heat treatment resulted in the formation of glass-ceramics, as indicated by the XRD analysis, which revealed a polycrystalline structure predominantly composed of a major phase of YAG (JCPDS card no:79–1891) [41] and a minor phase of Y_2_Si_2_O_7_ (JCPDS card no:42–0168) [41]. This suggests that, at this temperature, the glass powders underwent sufficient crystallization to form YAG and Y_2_Si_2_O_7_ phases, followed by sintering to produce dense and bulk glass-ceramics. According to the literature, Y_2_Si_2_O_7_ exhibits rich polymorphism and transforms into the δ-phase at high temperatures [41,42]. The crystalline phases detected in this study are generally consistent with those expected from the equilibrium phase diagram. YAS glass-ceramics can precipitate a variety of crystalline phases such as YAG, Al_6_Si_2_O_13_, Y_4_Al_2_O_9_, Y_2_SiO_5_ and Y_2_Si_2_O_7_, depending on the initial composition, as indicated by the phase diagram [39,40,43,44,45,46]. Among these, YAS glass-ceramics with YAG as the main crystalline phase are preferred due to their excellent plasma-corrosion resistance properties [20].

The phase fractions (%) were calculated using the intensity ratio method, based on the sum of the integrated intensities of all crystalline and amorphous phases present in the measured XRD patterns [47,48]. The analysis revealed 69.66% YAG phase, 27.36% Y_2_Si_2_O_7_ phase and 2.98% YAS amorphous (glass) phase.

Figure 2b shows an SEM (BSE mode) image of a typical YAS frit, revealing a dense microstructure. This figure shows mainly light gray grains with two different morphologies, both large and small. EDS elemental distribution analysis, shown in Figure 2c, revealed the presence of Y, Al, Si and O elements only. Based on the EDS point analysis (Figure S3), the large grains were identified as the YAG phase, while the small grains were identified as the Y_2_Si_2_O_7_ phase. EDS analysis indicated that the YAG phase consists of 16.37 %, 24.90 % and 58.73 % of Y, Al and O elements (at. %), respectively. In contrast, the Y_2_Si_2_O_7_ phase contains 19.28 %, 18.24 % and 62.48 % of Y, Si and O elements (at. %), respectively. The dark gray phase was identified as the YAS amorphous (glass) phase. These findings are consistent with the XRD data shown in Figure 2a.

**Figure 3:**
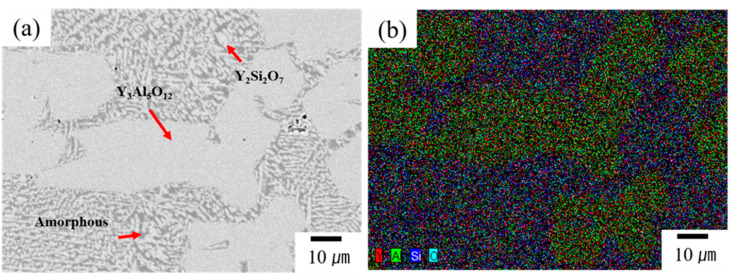
(**a**) BSE−SEM image of a typical YAS coating layer and (**b**) its corresponding EDS mapping image. No pores were found in the deposited coatings, indicating a dense microstructure. The image shows grains with two distinct contrasts and morphologies: dark gray and light gray, with varying sizes. According to the EDS mapping results, the light gray region with larger grain sizes is rich in Y, Al and O elements, while the light gray region with smaller grain sizes is rich in Y, Si and O elements.

BSE–SEM and EDS were used to examine the microstructure and elemental distribution of the YAS layer after coating on alumina, and the results are displayed in Figure 3. The BSE–SEM image of a typical YAS coating layer is presented in Figure 3a and the corresponding EDS mapping image is displayed in Figure 3b. No pores were found in the deposited coatings (Figure 3a), indicating a dense microstructure. The image shows grains with two distinct contrasts and morphologies: dark gray and light gray, with varying sizes. According to the EDS mapping results, the light gray region with larger grain sizes is rich in Y, Al and O elements, while the light gray region with smaller grain sizes is rich in Y, Si and O elements. Based on these EDS mapping data combined with XRD patterns, it was concluded that the gray phase with larger grain size corresponds to the YAG phase, the light gray phase with smaller grain size corresponds to the Y_2_Si_2_O_7_ phase and the minor dark gray phase corresponds to the amorphous (glass) YAS phase. The microstructure is similar to that of glass frit (Figure 2b), and its XRD analysis (Figure S4) also revealed major peaks of YAG phase and minor peaks of Y_2_Si_2_O_7_ phase. The phase fractions (%) were calculated to be 77.64% YAG phase, 13.68% Y_2_Si_2_O_7_ phase and 8.68% YAS amorphous (glass) phase. These phase categories and their distribution did not differ much from those of the glass frit before coating, and these results are in consistent with previous findings [41,44]. Minor variations in the phase fractions compared to the glass frit may be attributed to the altered mol% of Al_2_O_3_ content from the alumina substrate. From the above microstructural analyses, it has been confirmed that the two crystalline phases present in the YAS system are YAG and Y_2_Si_2_O_7_.

**Figure 4:**
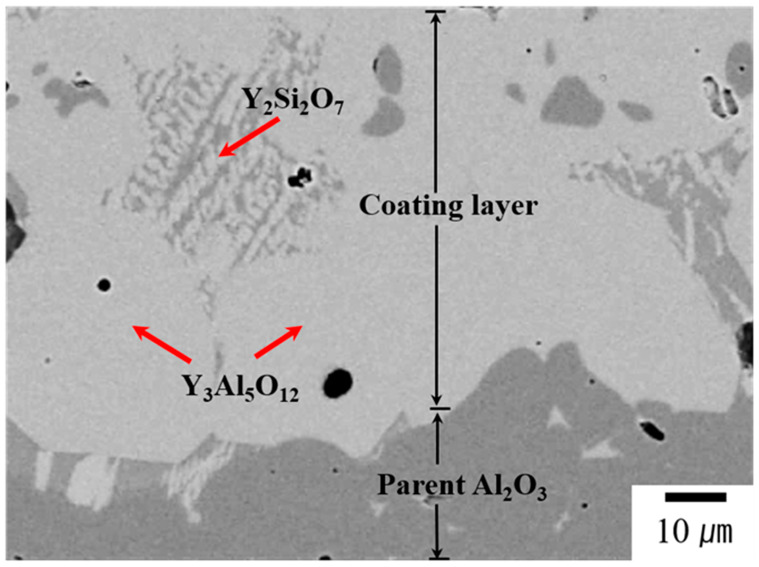
BSE–cross-sectional micrograph of YAS-coated alumina and it reveals a dense microstructure. Strong adhesion between the coating layer and the pristine alumina, without cracks, can be seen at the interface. Analyses revealed major peaks corresponding to YAG phase and minor peaks corresponding to Y_2_Si_2_O_7_ phase. These phase categories are identical to those observed in the YAS glass frit and coating layers.

Figure 4 shows a BSE–cross-sectional micrograph of YAS-coated alumina, which reveals a dense microstructure. The coating–substrate interface cross-sectional SEM image with the corresponding EDS elemental mapping is shown in Figure S5 and reveals that the coating layer has a thickness of 110 μm. Strong adhesion between the coating layer and the pristine alumina, without cracks, can be seen at the interface. XRD, SEM and EDS analyses revealed major peaks corresponding to the YAG phase and minor peaks corresponding to the Y_2_Si_2_O_7_ phase. These phase categories are identical to those observed in the YAS glass frit and coating layers.

**Figure 5:**
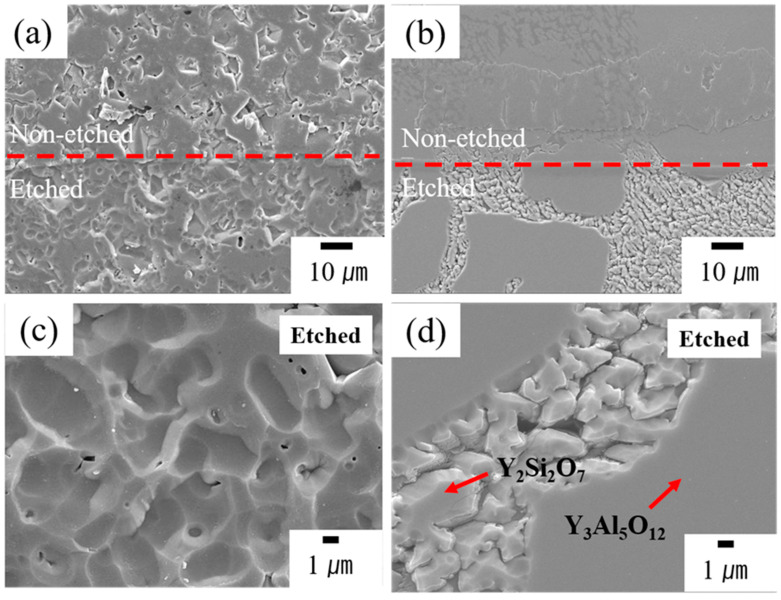
SEM images of the surface microstructures of both etched and non-etched regions of (**a**) uncoated (pristine) and (**b**) YAS-coated alumina ceramics post-exposure to plasma. No noticeable difference in the surface microstructure of YAS coatings was observed before and after exposure plasma exposure, compared to pristine alumina. High magnification surface SEM images after plasma treatment for (**c**) uncoated and (**d**) YAS-coated specimens.

The surface morphology of the specimens was examined using SEM to assess damage after plasma exposure. Figure 5 presents the SEM images of the surface microstructures of uncoated (pristine) and YAS-coated alumina ceramics post-exposure to plasma. Figure 5a,b compares both etched and non-etched regions. No noticeable difference in the surface microstructure of YAS coatings was observed before and after plasma exposure when compared to pristine alumina. High-magnification-surface SEM images after plasma treatment for uncoated and coated specimens are compared in Figure 5c,d, respectively. Many crater-like erosion sites were observed on the surface of the alumina (Figure 5c) following plasma exposure, whereas minimal surface damage was observed on the YAS-coated layer (Figure 5d). The high-Y region showed less plasma-induced erosion compared to the low-Y region. The surface morphological changes illustrated in this figure support this observation. According to the SEM images in Figure 5, the surface erosion of alumina was significant, while the YAS-coated layer demonstrated good etching resistance to plasma. It was observed that the surface of YAG phase appeared undamaged, but the material loss was relatively higher in the Y_2_Si_2_O_7_ phase.

Pristine alumina exhibited a noticeable etching loss of 0.0148% per square centimeter, whereas the YAS-coated layer showed a minimal loss of 0.0047% per square centimeter (about three times enhancement). The improved plasma-etching behavior of the YAS coating is likely due to the predominance of the YAG phase. It has been reported that materials containing Si react with F radicals in the plasma to form compounds with low sublimation points, which volatilize quickly. In contrast, the YAG phase reacts with F radicals to form fluorine compounds with very high sublimation points, resulting in a low etching rate [6]. Previous studies have shown that the YAG phase exhibits good resistance to fluorocarbon plasma corrosion, partly due to the rare-earth elements it contains, which inhibit reactions with fluorine-containing plasmas [20,49,50]. Additionally, the fluorides generated do not volatilize easily and form a protective layer on the ceramic surface, preventing further corrosion. Further investigations are required to analyze plasma etching behavior in more detail, particularly for the Y_2_Si_2_O_7_ phase.

## 4. Conclusions

In conclusion, the results indicate that YAS glass-ceramics, particularly with YAG as the predominant phase, exhibit excellent plasma-corrosion resistance. SEM and XRD analyses confirm the formation of YAG and Y_2_Si_2_O_7_ phases in the glass-ceramics, with microstructural analysis showing a dense, well-adhered coating on the alumina. Plasma exposure tests reveal that the YAS-coated alumina demonstrates minimal surface damage compared to uncoated alumina, highlighting the effectiveness of YAG in enhancing plasma resistance. These findings suggest that YAS coatings are highly effective for applications requiring resistance to plasma-induced corrosion. Further research is recommended to explore the detailed plasma etching behavior and optimize coating formulations for improved performance.

## Figures and Tables

**Table 1:** Plasma etching conditions used in the present work.

Parameter	Condition
Top RF power (W)	900
Bottom RF power (W)	200
C_4_F_6_ (Sccm)	30
Ar (Sccm)	60
O_2_ (Sccm)	15
Pressure (mTorr)	10
Operating time (h)	1

## Data Availability

The original contributions presented in the study are included in the article/Supplementary Material, further inquiries can be directed to the corresponding authors.

## Supplementary Materials

The following supporting information can be downloaded at: https://www.mdpi.com/article/10.3390/ma17184585/s1, Figure S1: Specimen’s configuration used for plasma exposure tests. Half of the specimen’s surface was masked with polyimide tape; Figure S2: PSA analysis of Y_2_O_3_, Al_2_O_3_ and SiO_2_ raw powders and it revealed average particle sizes of about 4.4, 2.7 and 0.7 μm, respectively; Figure S3: BSE SEM image and EDS analysis points of YAS frit. The large grains were identified as the YAG phase, while the small grains were identified as the Y_2_Si_2_O_7 _ phase. EDS analysis indicated that the YAG phase consists (Point A) of 16.37%, 24.90%, and 58.73% of Y, Al and O elements (at. %), respectively. In contrast, the Y_2_Si_2_O_7 _ (Point B) contains 19.28%, 18.24%, and 62.48% of Y, Si and O elements (at. %), respectively. The dark gray phase was identified as the YAS amorphous (glass) phase; Figure S4: XRD patterns of YAS-coated layer. The analysis revealed major peaks of YAG phase and minor peaks of Y_2_Si_2_O_7_ phase. The phase fractions (%) were calculated to be 77.64% YAG phase, 13.68% Y_2_Si_2_O_7 _ phase, and 8.68% YAS amorphous (glass) phase; Figure S5: BSE−cross-sectional micrograph of YAS-coated alumina and its elemental mapping.
