# Supplementary material for: Fabrication, Microstructure and Plasma Resistance Behavior of Y–Al–Si–O (YAS) Glass-Ceramics Coated on Alumina Ceramics
Source: Materials (Basel). 2024 Sep 18;17(18):4585. doi: 10.3390/ma17184585 (PMC11432973; doi:10.3390/ma17184585)
Supplement: Supplementary file 1 [file materials-17-04585-s001.zip › materials-3182121-supplementary.pdf]

Fabrication, Microstructure and Plasma Resistance Behavior of Y–Al–Si–O (YAS) Glass-Ceramics Coated on Alumina Ceramics

Eui Keun Park, Hwan-Yoon Jang, Seo-Yeon Jeon, Kati Raju * and Hyun-Kwuon Lee *

School of Advanced Materials Science and Engineering, Kumoh National Institute of Technology, Gumi 39177, Republic of Korea; czarkhan86@naver.com (E.K.P.); pray330@naver.com (H.-Y.J.); cilcis@naver.com (S.-Y.J.)

* Correspondence: author: katiraju@gmail.com (K.R.); hklee@kumoh.ac.kr (H.-K.L.); Tel: +82-54-478-7745 (H.-K.L.); Fax: +82-54-478-7769 (H.-K.L.)

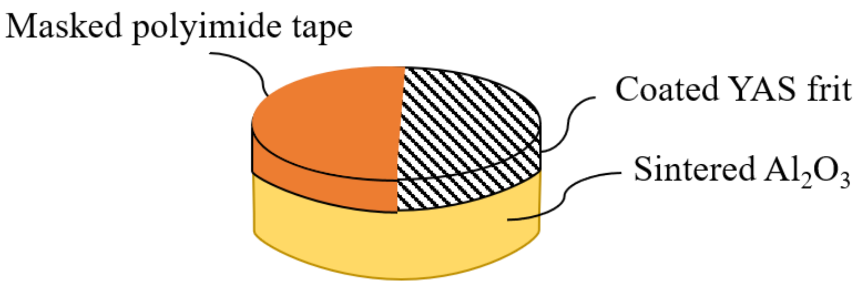

Figure S1. Specimen's configuration used for plasma exposure tests. Half of the specimen's surface was masked with polyimide tape.

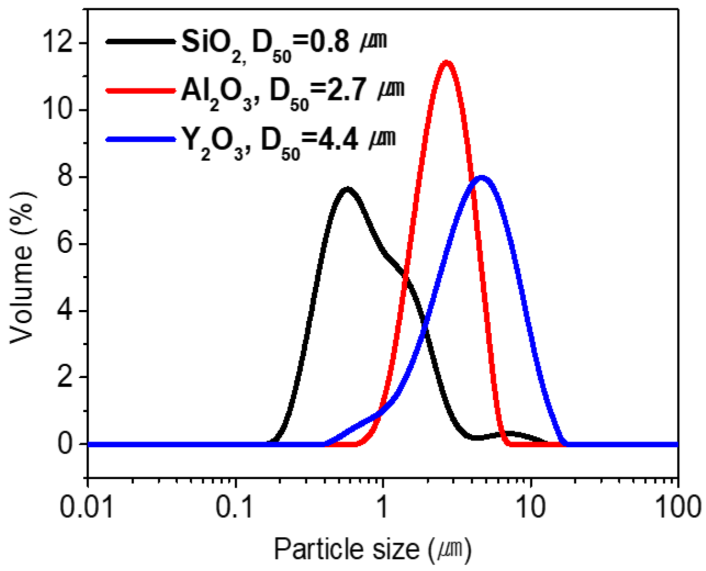

Figure S2. PSA analysis of Y_2O_3, Al_2O_3 and SiO_2 raw powders and it revealed average particle sizes of about 4.4, 2.7 and 0.7 µm, respectively.

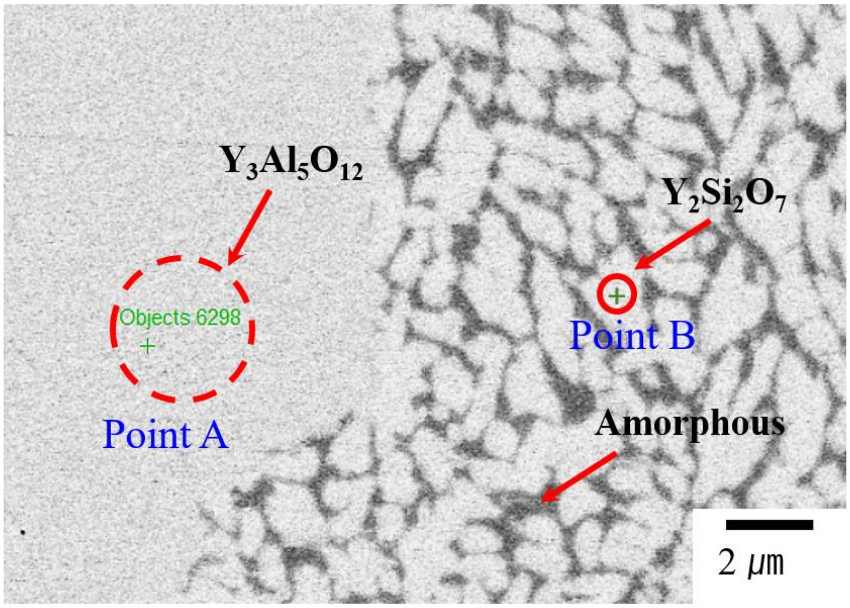

Figure S3. BSE SEM image and EDS analysis points of YAS frit. The large grains were identified as the YAG phase, while the small grains were identified as the $Y_2Si_2O_7$ phase. EDS analysis indicated that the YAG phase consists (Point A) of 16.37 %, 24.90 %, and 58.73 % of Y, Al and O elements (at. %), respectively. In contrast, the $Y_2Si_2O_7$ (Point B) contains 19.28 %, 18.24 %, and 62.48 % of Y, Si and O elements (at. %), respectively. The dark gray phase was identified as the YAS amorphous (glass) phase.

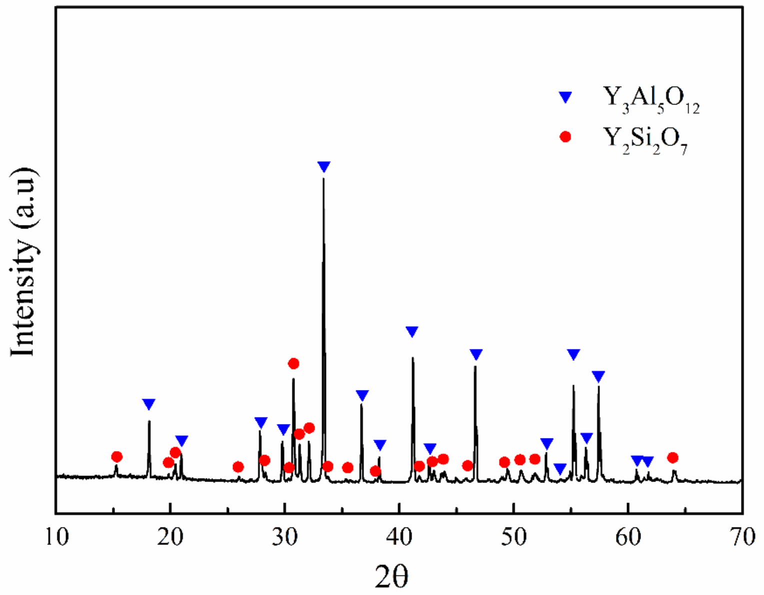

Figure S4. XRD patterns of YAS-coated layer. The analysis revealed major peaks of YAG phase and minor peaks of $Y_2Si_2O_7$ phase. The phase fractions (%) were calculated to be 77.64 % YAG phase, 13.68 % $Y_2Si_2O_7$ phase, and 8.68 % YAS amorphous (glass) phase.

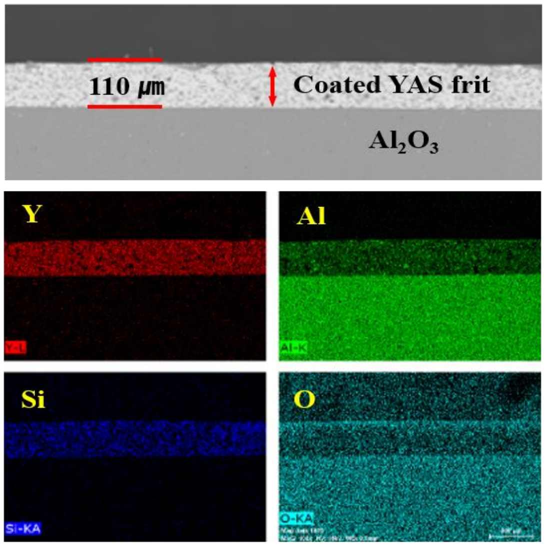

Figure S5. BSE−cross-sectional micrograph of YAS-coated alumina and its elemental mapping.
